# Synthesis and 3D LTCC Implementation of a Miniaturized Wide-Stopband Bandpass Filter Using Frequency-Dependent Coupling

**DOI:** 10.3390/mi17101173

**Published:** 2026-10-09

**Authors:** Hao Bai, Xiaoqi Yang, Jiali Wang, Jiawei Xu, Zhenguo Liu, Qiuyi Wu

**Affiliations:** 1 Frontiers Science Center for Flexible Electronics, Institute of Flexible Electronics, Northwestern Polytechnical University, Xi’an 710072, China; baihao504@mail.nwpu.edu.cn; 2 School of Electronic Engineering, Xidian University, Xi’an 710126, China; yangxiaoqi@stu.xidian.edu.cn (X.Y.);; 3 School of Artificial Intelligence, Xidian University, Xi’an 710126, China

**Keywords:** low-temperature co-fired ceramic (LTCC), bandpass filter, frequency-dependent coupling, transmission zero, wide stopband, miniaturization

## Abstract

This work presents an implementation-oriented synthesis methodology for a miniaturized wide-stopband bandpass filter in low-temperature co-fired ceramic (LTCC) technology. A fifth-order Chebyshev ladder prototype is first transformed to the bandpass domain, and frequency-dependent coupling (FDC) is introduced at the input and output resonators to generate near-band transmission zeros while preserving an inline topology. The inductive π networks are then converted into mutually coupled inductor equivalents that can be implemented by vertically overlapping metal traces in the multilayer LTCC stack, reducing the need for extreme lumped-element values. Auxiliary parallel LC resonators are further placed at the input/output ports as FDC pole units to create additional high-frequency transmission zeros and enlarge the far-stopband design margin. A 3–5 GHz prototype centered at 4 GHz was fabricated in a 4 mm × 2.8 mm × 1 mm package. Measurements confirm transmission zeros near 2 and 6 GHz and more than 30 dB rejection over DC–2 GHz and 6–18 GHz. The results demonstrate the feasibility of the proposed LTCC-oriented design methodology.

## 1. Introduction

The continuing integration of sub-6 GHz radio-frequency (RF) front ends requires bandpass filters that simultaneously provide a small footprint, low in-band loss, sharp transition bands, and strong suppression of harmonic and spurious responses. These requirements are difficult to satisfy in a single miniaturized implementation because the mechanisms used to increase selectivity or broaden the stopband often introduce additional resonators, cross-couplings, or extreme element values that complicate fabrication and increase sensitivity to parasitic effects.

Low-temperature co-fired ceramic (LTCC) technology is attractive for compact RF filters because its multilayer three-dimensional (3D) architecture allows capacitors, inductors, vias, and electromagnetic coupling structures to be vertically integrated within a small package [1]. Previous LTCC bandpass filters have used stepped-impedance stubs to control transmission zeros (TZs) and bandwidth [2], cross-coupled resonators to improve selectivity [3], and circuit-to-layout mapping techniques to reduce the burden of full-wave optimization [4]. However, in strongly miniaturized LTCC networks, the self-resonance of spiral inductors, interlayer capacitance, and unintended coupling between adjacent stages can introduce high-frequency spurious responses and reduce the usable stopband [5].

Transmission zeros can be synthesized using coupling-matrix cross-coupling [6], extracted-pole techniques [7], or frequency-dependent coupling (FDC) [8]. FDC is particularly attractive for inline or ladder topologies because controllable TZs can be introduced without the routing complexity of conventional cross-coupled structures. Wide-stopband responses have also been achieved by tailoring resonator modes and suppressing high-order resonances [9]. Recent compact filters further demonstrate the usefulness of heterogeneous LTCC resonators [10,11,12,13], integrated passive-device (IPD) coupling schemes [14], hybrid wide-stopband resonators [15], synthesis-oriented miniaturized filtering structures [16], and 3D stacked coupling architectures [17].

Building on the established frequency-dependent coupling (FDC) synthesis framework [8], this work develops an LTCC-oriented synthesis and implementation methodology for miniaturized bandpass filters with enhanced selectivity and extended stopband suppression. Starting from a fifth-order ladder prototype, FDC sections are incorporated into the input and output resonators to generate a pair of controllable transmission zeros (TZs) near the passband edges while preserving a simple inline topology. To facilitate a compact and physically realizable LTCC implementation, the inductive π-networks associated with the FDC sections are transformed into equivalent mutually coupled-inductor networks, enabling the required coupling to be implemented through the vertical overlap of metal traces in the multilayer LTCC structure. Furthermore, auxiliary parallel LC resonators are introduced at the input and output ports as additional FDC pole units to generate high-frequency TZs and suppress parasitic responses in the upper stopband. Based on this synthesis-to-implementation procedure, a fifth-order LTCC bandpass filter operating over 3–5 GHz is designed, fabricated, and measured. The agreement between the simulated and measured responses validates the proposed methodology and demonstrates its capability to simultaneously achieve compact implementation, high selectivity, and wide-stopband suppression.

## 2. Filter Synthesis and Design Methodology

### 2.1. Low-Pass Prototype and FIR Absorption

The synthesis starts from a normalized fifth-order Chebyshev low-pass ladder prototype. During the low-pass-to-bandpass transformation, a frequency-invariant reactance (FIR) may appear in the transformed network. Because an FIR is an idealized element that cannot be implemented directly by conventional passive components, it is absorbed into an adjacent parallel LC resonator through a narrowband susceptance-slope transformation. For the resonant branch containing the FIR, the admittance can be written as

(1) $$Y=\mathrm{j\omega C}+\frac{1}{\mathrm{j\omega L}}+\mathrm{jB}$$

where *C* and *L* are the equivalent capacitance and inductance, respectively, *B* is the frequency-invariant susceptance, and *ω* is the angular frequency. The susceptance slope at the resonant angular frequency *ω*_0_ is

(2) $$b=\frac{\omega_{0}}{2}(C+\frac{1}{\omega_{0}^{2}L})$$

By selecting a parallel LC resonator that preserves both the resonant frequency and the susceptance slope, the non-physical FIR term is absorbed into a realizable LC network. An inductively coupled ladder is then selected as the baseline topology because it is compatible with multilayer LTCC implementation and provides a convenient route for subsequent FDC transformation.

### 2.2. FDC-Based Transmission-Zero Generation

**Figure 1:**
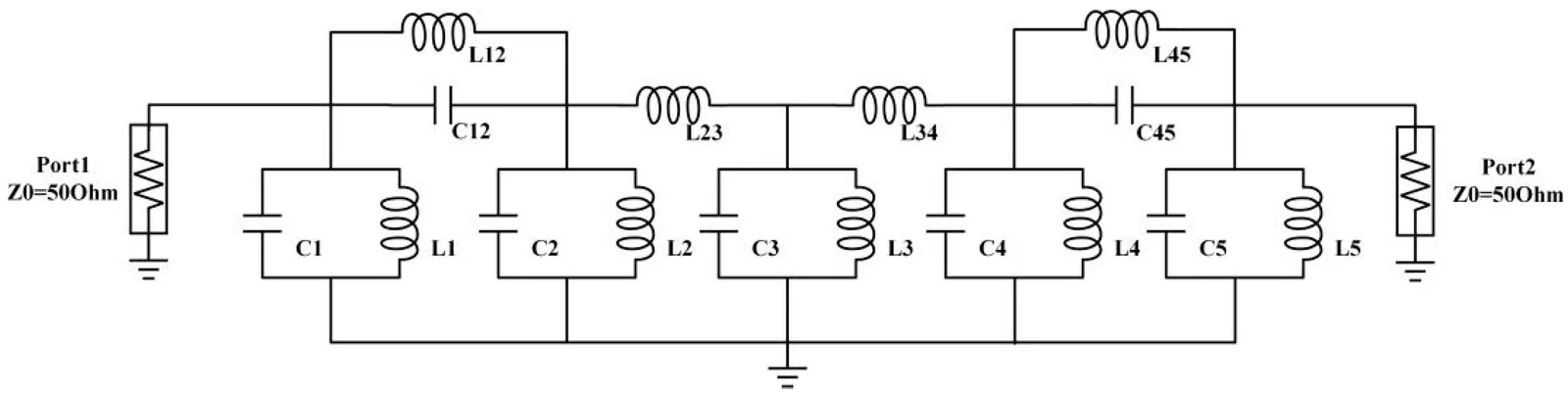
Inductively coupled fifth-order prototype circuit after FIR absorption and introduction of the near-band FDC sections.

To sharpen the lower and upper passband skirts, FDC sections are introduced around the first and last resonators. The underlying TZ mechanism can be understood from the resonant cancellation of a frequency-dependent branch: a series resonance embedded in a shunt path can create a low-impedance cancellation condition, whereas a parallel resonance embedded in a series path can create a high-impedance condition. In the present design, the FDC elements are selected so that the two principal near-band TZs are located at approximately 2 and 6 GHz, on either side of the 3–5 GHz passband. Figure 1 shows the resulting inductively coupled principal circuit.

For the design example, a fifth-order Chebyshev response with a center frequency of 4 GHz, a fractional bandwidth of 50%, and an in-band return loss of approximately 20 dB is adopted. The FDC sections at the input and output resonators are synthesized to locate the two principal transmission zeros near 2 and 6 GHz, respectively. The circuit parameters obtained from the low-pass-to-bandpass transformation and the subsequent FDC and mutual-inductor transformations are used as the initial values for the LTCC physical implementation, while full-wave EM optimization is employed to compensate for parasitic effects introduced by the multilayer structure and to complete the physical mapping.

To make the numerical starting point of the synthesis explicit, the specified 20 dB in-band return loss corresponds to a Chebyshev ripple of 0.04365 dB and a ripple factor ε = 0.10050. For N = 5, the normalized low-pass prototype values are *g*_0_ = 1, *g*_1_ = 0.97323, *g*_2_ = 1.37228, *g*_3_ = 1.80319, *g*_4_ = 1.37228, *g*_5_ = 0.97323, and *g*_6_ = 1. Using *R*_0_ = 50 Ω, *f*_0_ = 4 GHz, and FBW = 0.50, the standard low-pass-to-bandpass transformation gives the reference element values listed in Table 1. These values define the numerical starting point before FIR absorption and the subsequent FDC and mutually coupled-inductor transformations.

### 2.3. Mutually Coupled-Inductor Equivalent for 3D LTCC Realization

**Figure 2:**
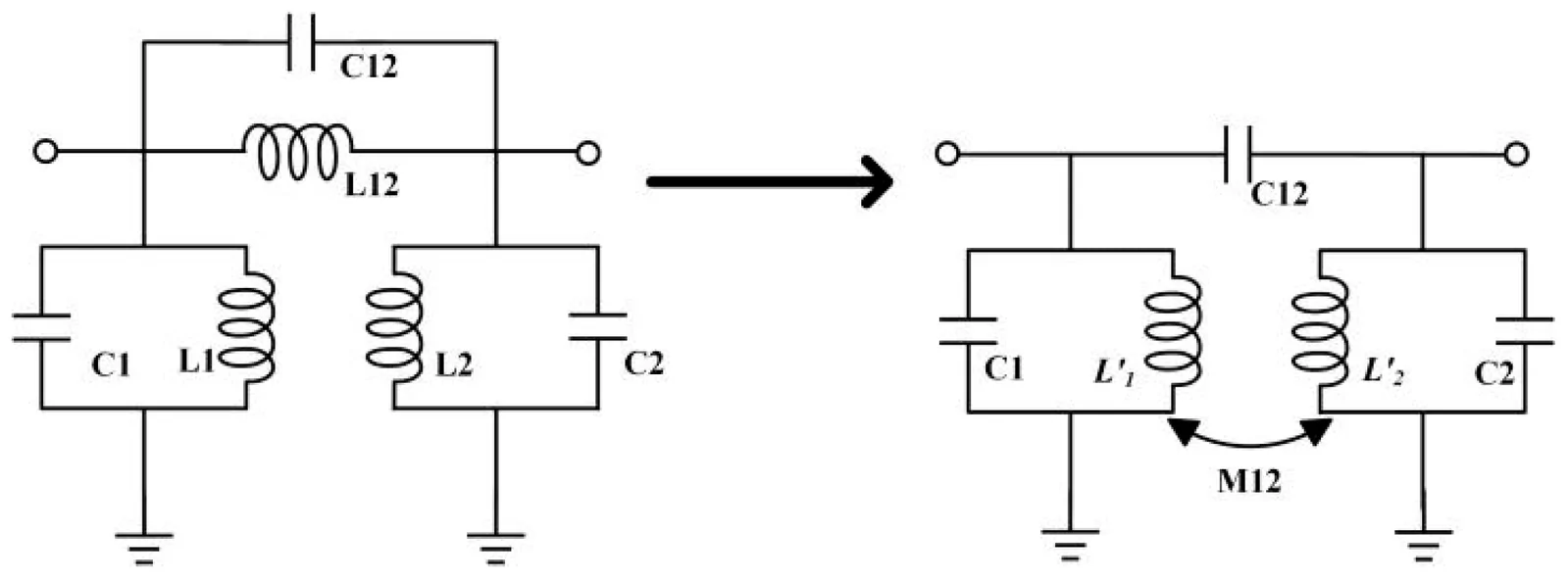
Equivalent transformation of the inductive coupling section, showing the conversion of the inductive π network in the principal circuit into a mutually coupled-inductor representation.

Direct implementation of the inductive π network in Figure 1 can require element values that are difficult to realize compactly and can aggravate high-frequency non-lumped effects. To obtain an LTCC-friendly representation, each relevant inductive π network is first transformed into an equivalent T network and then into a pair of mutually coupled inductors, as illustrated in Figure 2. This transformation preserves the circuit response while replacing a series inductive branch with magnetic coupling that can be generated physically by vertically overlapping inductor traces.

*L*_1_*, L*_2_, and *L*_12_ denote the inductances in the original inductive network of Figure 2. The equivalent mutual inductance *M* and the transformed self-inductances *L*_1_*′* and *L*_2_*′* are calculated as

(3) $$M=\frac{L_{1}L_{2}}{L_{1}+L_{2}+L_{12}},{L_{1}}^{\prime }=\frac{L_{1}(L_{2}+L_{12})}{L_{1}+L_{2}+L_{12}},{L_{2}}^{\prime }=\frac{L_{2}(L_{1}+L_{12})}{L_{1}+L_{2}+L_{12}}$$

**Figure 3:**
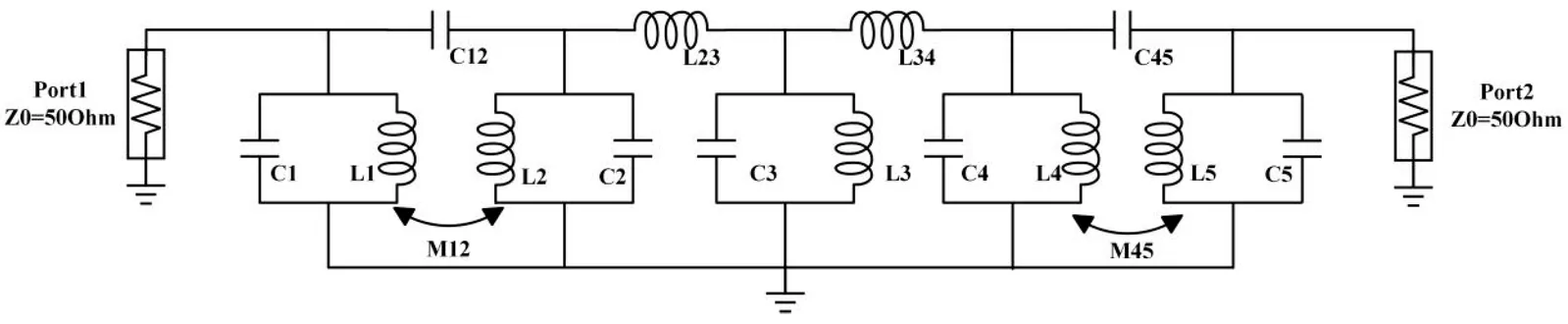
Prototype circuit after replacing the inductive π networks with mutually coupled-inductor equivalents M12 and M45.

The transformed prototype, including the two mutually coupled sections M12 and M45, is shown in Figure 3. In the 3D LTCC implementation, these mutual inductances are generated by the vertical overlap of adjacent spiral or planar traces. This mapping reduces the number of isolated lumped inductors, balances the required element values, and uses the vertical dimension of the ceramic stack to reduce the lateral footprint.

For practical implementation, the synthesis-to-layout procedure proceeds as follows. The ladder prototype is first determined from the specified center frequency, fractional bandwidth, and return loss. After the bandpass transformation, the FIR terms are absorbed into the corresponding network parameters, and the FDC branches are synthesized to realize the prescribed near-band transmission zeros (TZs). The resulting inductive π networks are then converted into equivalent mutually coupled-inductor representations, after which the lumped and coupled elements are mapped to the multilayer LTCC geometry. Full-wave electromagnetic (EM) optimization is subsequently employed for parasitic-effect compensation and final physical refinement.

The transformation in Equation (3) is an exact algebraic two-port equivalence. Substitution of *M*, *L*_1_′, and *L*_2_′ into the transformed network preserves the driving-point and transfer functions of the original lumped network. Consequently, prior to physical mapping, the two representations have identical S-parameters over frequency. The coupling coefficient for the input section is

(4) $$k_{12}=\frac{M_{12}}{\sqrt{{L_{1}}^{\prime }{L_{2}}^{\prime }}}=\sqrt{\frac{L_{1}L_{2}}{\left(L_{2}+L_{12}\right)\left(L_{1}+L_{12}\right)}}$$

with the analogous expression applied to the *L*_4_–*L*_5_ section. The nominal fifth-order synthesis targets are *k*_12_ = *k*_45_ = 0.43265 and *k*_23_ = *k*_34_ = 0.31785. In the LTCC implementation, the corresponding magnetic coupling is realized by the vertical overlap of the conductor traces and evaluated in the three-dimensional full-wave model.

## 3. Wide-Stopband Enhancement and LTCC Implementation

### 3.1. Parasitic Effects and Stopband Design Margin

When the synthesized circuit is mapped to the multilayer LTCC structure, the complete HFSS model includes all metal traces, vias, dielectric layers, and their electromagnetic interactions. It therefore captures spiral-inductor self-capacitance, interlayer and inter-conductor capacitance, and unintended interstage coupling, including the L3 and L4/L5 regions, which are absent from the ideal lumped-element model. The initial full-wave model exhibits an additional transmission feature near 7.3 GHz. A first-order equivalent description of this characteristic frequency is

(5) $$f_{p}=\frac{1}{2\pi \sqrt{L_{p}C_{p}}}$$

which gives L_p_C_p_ = 0.475329 nH·pF at 7.3 GHz. This equivalent constraint is consistent with a distributed parasitic mode arising from the combined effects of spiral self-capacitance, interlayer capacitance, and unintended electromagnetic coupling. The auxiliary FDC units are therefore used to suppress the transmitted response associated with this mode rather than to imply physical elimination of the underlying distributed resonance.

### 3.2. Auxiliary FDC Pole Units for Far-Stopband Transmission Zeros

**Figure 4:**
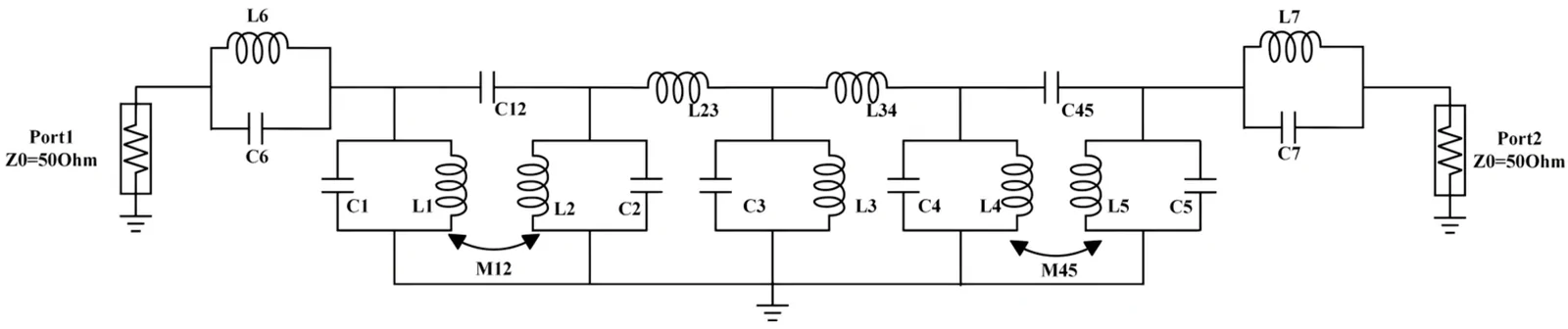
Wide-stopband prototype circuit including the auxiliary parallel LC FDC pole units at the input and output ports.

To restore the upper-stopband attenuation margin, auxiliary parallel LC resonators are introduced at the input and output ports as additional FDC pole units, as illustrated in Figure 4. The auxiliary branches, consisting of L_6_–C_6_ and L_7_–C_7_, respectively, are incorporated into the signal path while retaining the original near-band FDC sections and mutually coupled-inductor network. At their respective parallel-resonance frequencies, these branches present high impedances, thereby introducing additional transmission zeros in the upper stopband. For an ideal parallel LC branch placed in the series signal path, the transmission-zero frequency is governed by the resonance condition. For an ideal parallel LC branch placed in the series signal path, the transmission-zero frequency is governed by the resonance condition.

(6) $$f_{z}=\frac{1}{2\pi \sqrt{\mathrm{LC}}}$$

The prescribed zero frequencies therefore determine the LC products summarized in Table 2. The LC product primarily fixes the transmission-zero location, whereas the individual L and C values also determine the off-resonant branch impedance and are selected jointly with the realizable LTCC geometry and its parasitic effects. In the optimized structure, the auxiliary branches generate suppression notches near 11 and 17 GHz while the principal near-band transmission zeros remain close to 2 and 6 GHz. 

### 3.3. LTCC Physical Implementation and Full-Wave Optimization

**Figure 5:**
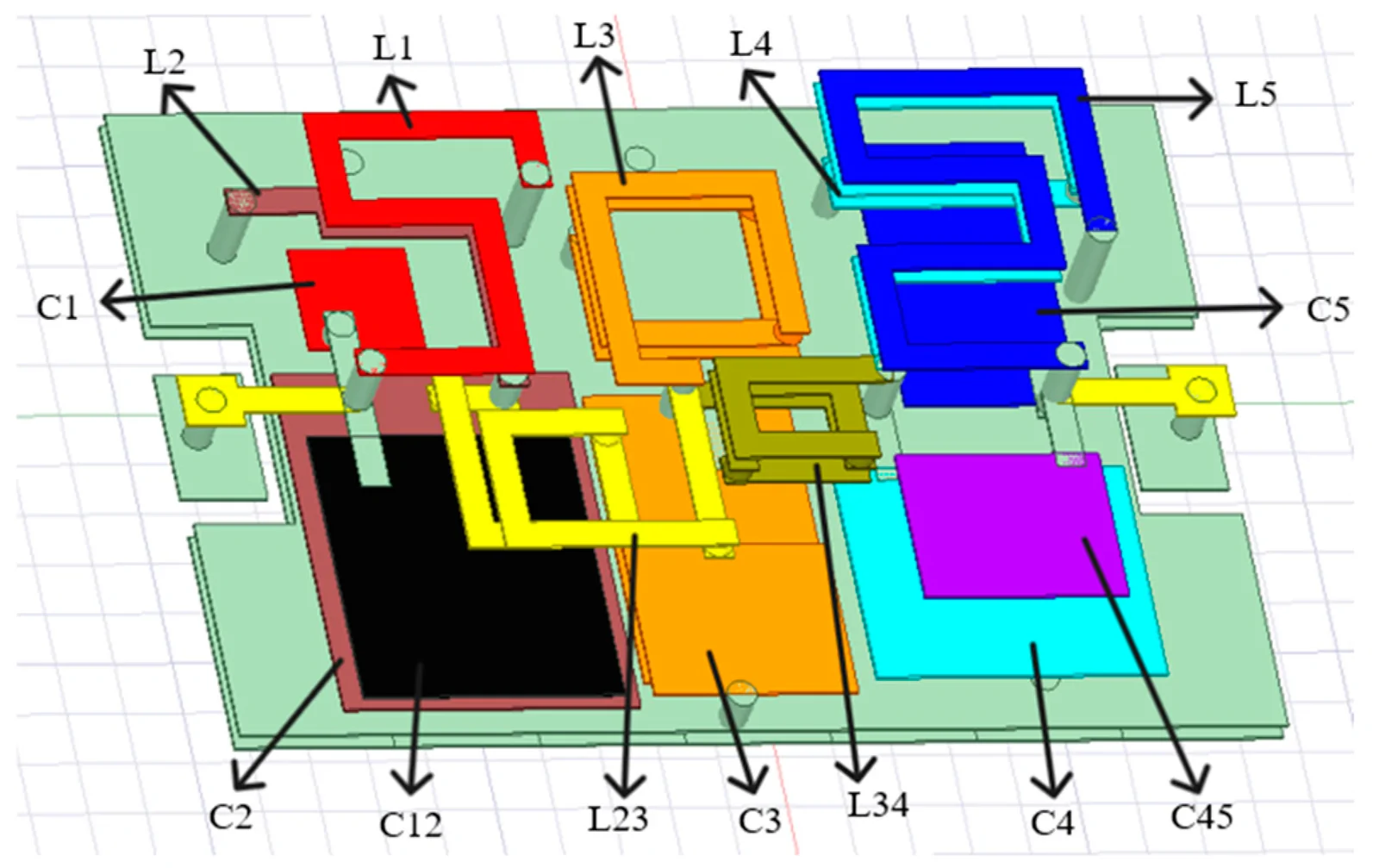
Three-dimensional modular layout of the proposed LTCC filter and the locations of the principal lumped and coupling elements.

A 3–5 GHz bandpass filter with a center frequency of 4 GHz is designed as a representative example. The filter is implemented using an independently developed forsterite (Mg_2_SiO_4_)-based LTCC ceramic with a relative permittivity of 7.0, a dielectric loss tangent of 0.002, and an individual layer thickness of 60 μm. The active filtering network occupies an approximate footprint of 2.8 mm × 2.3 mm in the x–y plane and is integrated within a packaged volume of 4 mm × 2.8 mm × 1 mm. The three-dimensional arrangement of the constituent components is illustrated in Figure 5. Most capacitors are realized using metal–insulator–metal (MIM) structures, while C_3_ adopts a custom-designed vertically interdigitated capacitor (VIC) topology to accommodate the required capacitance within the available multilayer volume. The inductive elements are realized as planar or multilayer spiral inductors, with the specific configuration selected according to the required inductance values, coupling characteristics, and available physical space.

**Figure 6:**
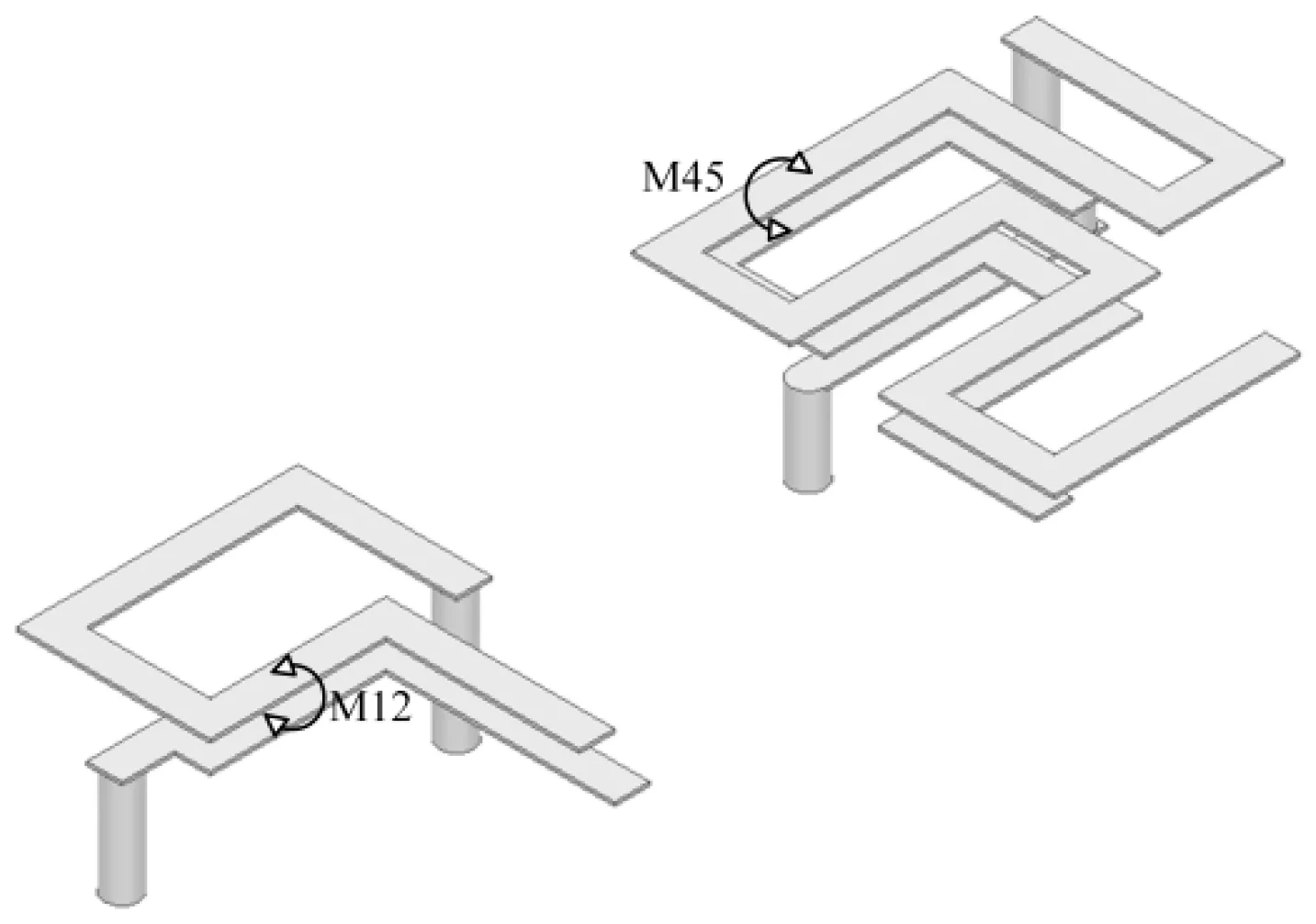
Three-dimensional realization of the principal mutual-coupling regions M12 and M45 by vertically overlapping LTCC inductor traces.

The two mutual-coupling sections are implemented by vertically overlapping metal traces, as shown in Figure 6. The overlap region provides a direct physical tuning parameter for M12 and M45 and allows the magnetic coupling to be strengthened or weakened without substantially increasing the lateral area.

**Figure 7:**
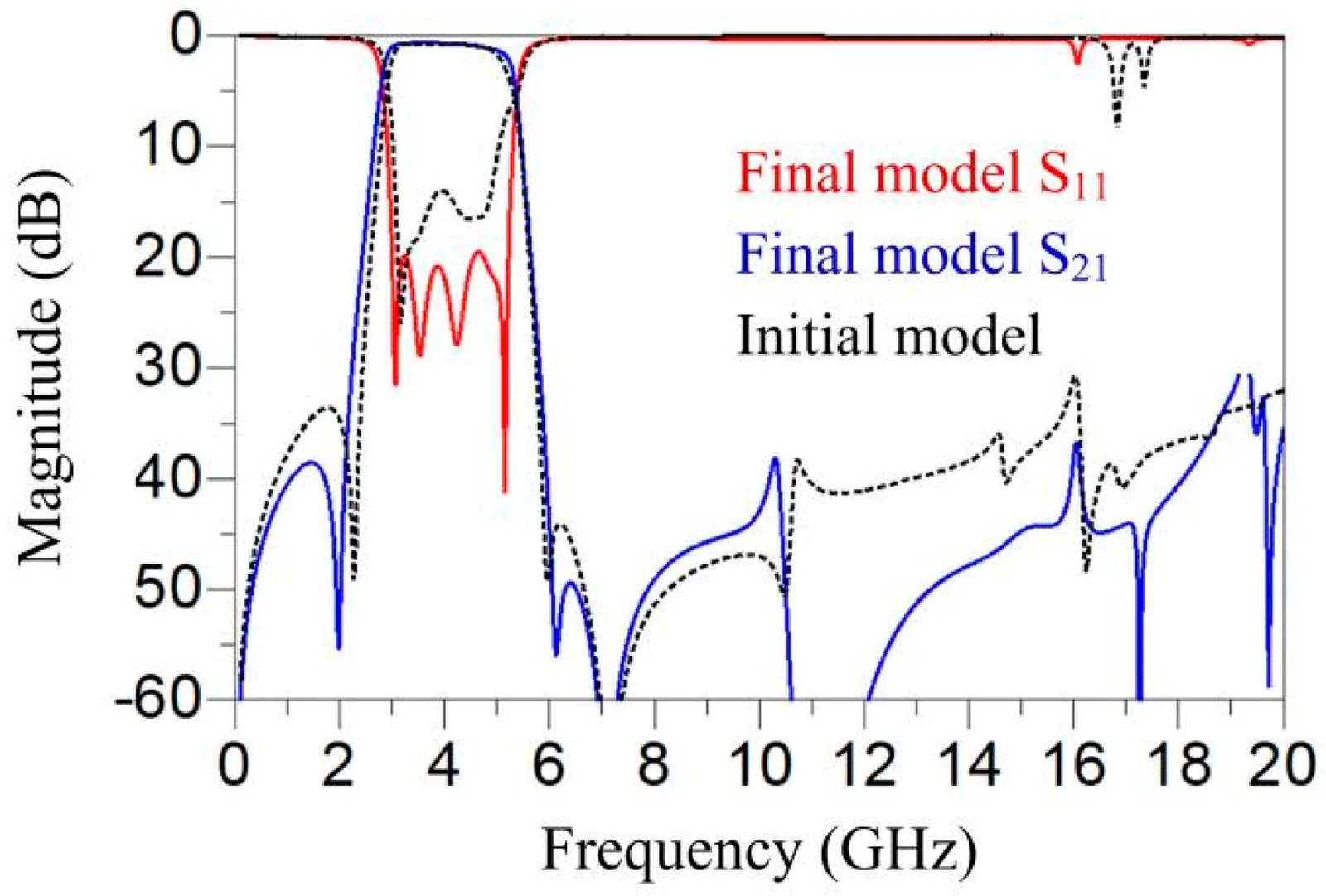
Full-wave EM responses of the initial and final LTCC models. The auxiliary FDC pole units increase the high-frequency attenuation margin while preserving the 3–5 GHz passband.

Full-wave EM optimization is performed in Ansys HFSS using the complete multilayer structure, with the fixed material and fabrication parameters summarized in Table 3. The synthesis stage defines the electrical topology, resonant frequencies, and coupling targets, whereas the full-wave stage maps these targets to realizable line lengths, overlap areas, and capacitor geometries under the LTCC fabrication constraints. The constrained optimization proceeds as follows: the synthesized elements are first mapped to MIM, VIC, spiral, and vertically coupled structures; the 3–5 GHz passband and near-band transmission zeros are aligned through resonator dimensions and the M12/M45 overlap regions; the initial structure is then evaluated over the extended stopband; the auxiliary FDC pole units are introduced to restore attenuation margin around the 7.3 GHz parasitic feature; and a final local geometric refinement is performed while preserving the synthesized passband topology. After this procedure, the near-band zeros remain at approximately 2 and 6 GHz, additional suppression notches occur near 11 and 17 GHz, and the optimized simulation provides better than 35 dB rejection over 6–18 GHz, as shown in Figure 7.

### 3.4. First-Order Sensitivity to Fabrication-Related Variations

To assess the robustness of the proposed LTCC implementation against fabrication-related variations, a normalized first-order sensitivity analysis is performed. The analysis is based on the resonant-frequency, MIM-capacitance, and magnetic-coupling relations

f ∝ (LC)^−1/2^ (7)

(8) $$C\propto \frac{\varepsilon_{r}}{t}$$

(9) $$k=\frac{M}{{\left(L_{1}L_{2}\right)}^{1/2}}$$

A uniform ±5% perturbation is used as a common normalized basis for comparing the electrical sensitivities of the principal parameters. A +5% variation in L, C, or ε_r_ produces an approximately 2.41% downward shift in resonance frequency, whereas a −5% variation produces an approximately 2.60% upward shift. For the dielectric thickness, C ∝ 1/t gives frequency shifts of approximately +2.47% and −2.53% for +5% and −5% variations, respectively. A ±5% variation in mutual inductance M, which represents the first-order electrical effect of overlap and interlayer-alignment variation, produces a corresponding ±5% change in the coupling coefficient. Simultaneous ±5% variations in both self-inductances with M fixed change the coupling coefficient by approximately −4.76% and +5.26%, respectively. Conductor-width variations primarily influence the effective inductance and conductor loss and are represented at first order by the normalized inductance perturbation. The resulting sensitivities are summarized in Table 4.

## 4. Fabrication and Experimental Validation

### 4.1. Sample Fabrication and Test Fixture

**Figure 8:**
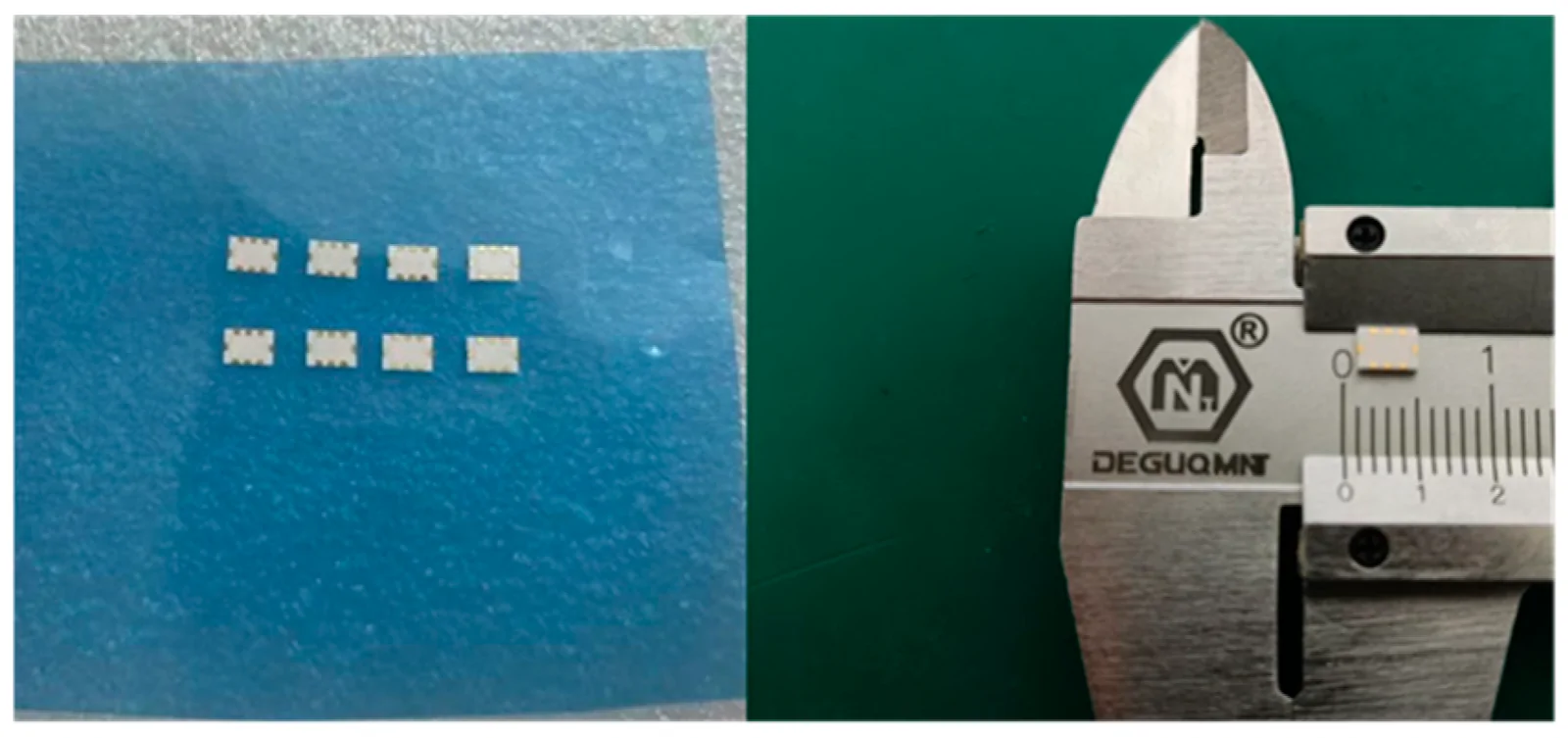
Photograph of the fabricated LTCC devices and dimensional measurement of the packaged device.

The proposed LTCC filter was fabricated using a standard multilayer cofiring process. The packaged dimensions are 4 mm × 2.8 mm × 1 mm, and the external terminals are finished with electroplated gold. Figure 8 shows the fabricated devices and the dimensional measurement. The active filtering network occupies approximately 2.8 mm × 2.3 mm, whereas the overall packaged device measures 4 mm × 2.8 mm × 1 mm.

For measurement, a coplanar-waveguide (CPW) test board with two SMA connectors is fabricated on Rogers 4350. The CPW signal-line width and gap are 0.49 and 0.65 mm, respectively, corresponding to a calculated characteristic impedance of approximately 49.9 Ω. Relative to a 50 Ω system, this corresponds to |Γ| ≈ 0.0010, or a return loss of approximately 60 dB, indicating that the nominal line-impedance mismatch is negligible. A compensation slot is introduced at the connector-to-CPW transition to reduce the local discontinuity and improve the high-frequency match. The LTCC filter is soldered to the CPW fixture and characterized using a vector network analyzer (E5080B, Keysight Technologies, Santa Rosa, CA, USA). Fixture de-embedding is not applied; consequently, the measurement reference planes remain at the fixture ports and the reported response includes CPW propagation loss together with the electrical effects of the SMA/CPW transitions, contact pads, and soldered device interfaces.

### 4.2. Measured Filter Response

**Figure 9:**
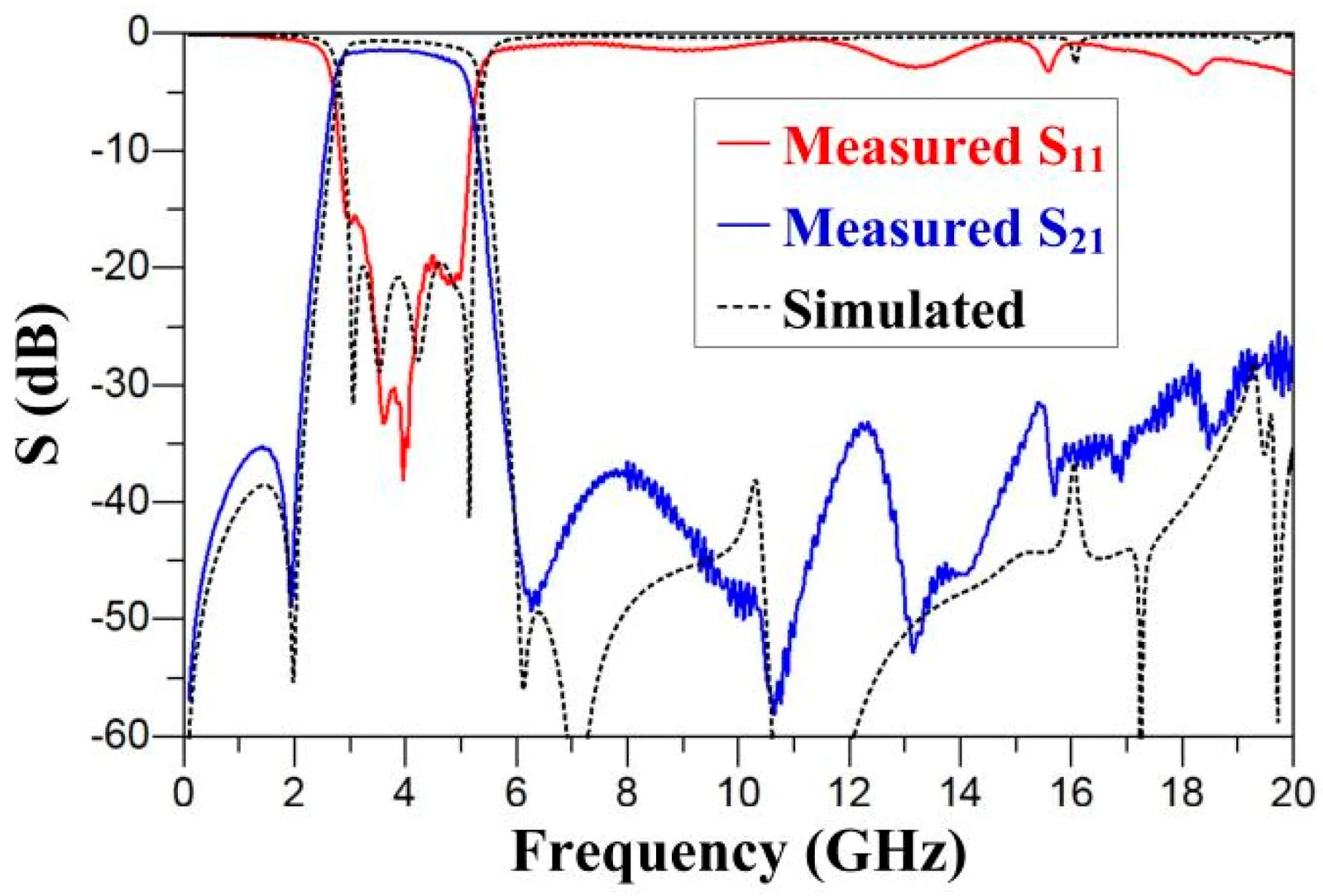
Simulated and measured S-parameters of the fabricated LTCC filter.

Figure 9 compares the fixture-inclusive measured S-parameters with the device-only full-wave simulation. The measured passband and the two intended near-band transmission zeros around 2 and 6 GHz agree reasonably well with the full-wave prediction. More than 30 dB rejection is maintained over the measured lower stopband from DC to 2 GHz and the upper stopband from 6 to 18 GHz. With *f*_0_ = 4 GHz, the demonstrated upper stopband therefore extends to approximately 4.5 *f*_0_ at the 30 dB rejection level.

Local differences between the simulated and measured S21 curves should not be interpreted as a direct change in intrinsic filter loss because the two responses are referenced to different planes. A small displacement of a passband edge or transmission zero can produce a locally higher measured |S21| at a fixed frequency even when the overall measured insertion loss is not lower than the simulated value. Because the measurement reference planes are located at the fixture ports, the observed discrepancy reflects the combined influence of CPW propagation loss, connector/CPW and soldered-device transitions, and LTCC fabrication tolerances. No unique quantitative partition is assigned to these contributions. A direct intrinsic-loss comparison requires transfer of the calibration reference planes to the device terminals through fixture de-embedding or equivalent fixture characterization. Improved transition repeatability, reduced interface discontinuity, higher conductor conductivity, sufficient conductor thickness, and tighter process control would further reduce measurement uncertainty and intrinsic loss.

The measurements reported in this work were performed under small-signal excitation and are intended to validate the frequency-selective characteristics of the proposed LTCC filter. The maximum RF power-handling capability was not evaluated in the present study. At elevated power levels, local electric-field enhancement, conductor heating, current crowding, and the dielectric strength of the LTCC material may influence the filter response and ultimately limit the allowable input power. Quantitative assessment of these effects requires dedicated high-power measurements and electrothermal characterization and is beyond the scope of the present small-signal validation.

Figure 8 shows several fabricated devices, whereas Figure 9 presents the measured response of a representative fixture-mounted device. The present experimental characterization is therefore interpreted as device-level validation of the proposed synthesis and LTCC implementation methodology rather than as a batch-statistical assessment. Confidence intervals are not assigned to the representative S-parameter response; a batch repeatability analysis requires identical calibrated measurements of multiple devices followed by statistical evaluation of the principal filter characteristics.

### 4.3. Performance Comparison with Reported Filters

Table 5 compares the proposed filter with representative LTCC- and IPD-based bandpass filters reported in the literature. The comparison includes implementation technology, filter order, center frequency, 3 dB fractional bandwidth, insertion loss, number of transmission zeros, stopband rejection and normalized extent, normalized footprint, and principal implementation characteristics. Quantities not stated in the cited publications are denoted as N/R.

Overall, the proposed filter provides a practical trade-off among bandwidth, selectivity, stopband suppression, footprint, and implementation complexity. The synthesis-stage contributions comprise the fifth-order ladder prototype, the generation of the near-band transmission zeros through frequency-dependent coupling, and the transformation of the inductive π-networks into mutually coupled-inductor equivalents. The auxiliary FDC pole units and final full-wave geometric refinement are implementation-stage measures used to compensate for distributed LTCC parasitic effects and to recover the intended upper-stopband attenuation. This distinction separates the analytical origin of the passband and near-band transmission-zero topology from the electromagnetic corrections required for the three-dimensional LTCC realization.

## 5. Conclusions

This work presents an LTCC-oriented synthesis-to-implementation methodology for a miniaturized wide-stopband bandpass filter. Starting from a fifth-order Chebyshev ladder prototype, frequency-dependent coupling (FDC) is introduced at the input and output resonators to generate near-band transmission zeros while maintaining a simple inline topology. The resulting inductive π-networks are transformed into equivalent mutually coupled-inductor networks, enabling compact realization through vertically overlapped metal traces in the multilayer LTCC structure. Auxiliary FDC pole units are further incorporated at the input and output ports to introduce additional high-frequency transmission zeros and extend the upper stopband. A 3–5 GHz prototype was fabricated in a compact 4 mm × 2.8 mm × 1 mm package. The measured results exhibit transmission zeros near 2 and 6 GHz and out-of-band rejection exceeding 30 dB over DC–2 GHz and 6–18 GHz, corresponding to an upper stopband extension of approximately 4.5 *f*_0_. The good agreement between simulation and measurement validates the proposed design procedure and demonstrates the applicability of the proposed methodology to compact LTCC filters requiring high selectivity and broad stopband suppression. The reported measurement constitutes a small-signal, fixture-inclusive validation of a representative device; high-power operation and batch-level statistical repeatability are outside the scope of the present experimental characterization.

## Figures and Tables

**Table 1:** Numerical starting point derived from the specified fifth-order Chebyshev response.

Quantity	Prototype Value/Expression	L (nH)	C (pF)	Remark
Normalized prototype	*g*_0_ = 1; *g*_1_ = 0.97323; *g*_2_ = 1.37228; *g*_3_ = 1.80319; *g*_4_ = 1.37228; *g*_5_ = 0.97323; *g*_6_ = 1	—	—	RL = 20 dB; ripple = 0.04365 dB; ε = 0.10050
Reference BPF resonator 1	*g*_1_ = 0.97323	3.87235	0.408833	series reference resonator
Reference BPF resonator 2	*g*_2_ = 1.37228	0.72487	2.184048	shunt reference resonator
Reference BPF resonator 3	*g*_3_ = 1.80319	7.17466	0.220658	series reference resonator
Reference BPF resonator 4	*g*_4_ = 1.37228	0.72487	2.184048	shunt reference resonator
Reference BPF resonator 5	*g*_5_ = 0.97323	3.87235	0.408833	series reference resonator
Nominal adjacent coupling	*k*_ij_ = FBW/√(*g*_i_*g*_j_)	—	—	*k*_12_ = *k*_45_ = 0.43265; *k*_23_ = *k*_34_ = 0.31785
Nominal external Q	*Q*_e_ = *g*_0_*g*_1_/FBW	—	—	*Q*_e1_ = *Q*_e5_ = 1.94646

**Table 2:** Resonance constraints for the near-band and auxiliary FDC branches.

Branch	Target Zero (GHz)	LC Product (nH·pF)	Function
*L*_12_–*C*_12_	2	6.332574	Lower near-band transmission zero
*L*_45_–*C*_45_	6	0.703619	Upper near-band transmission zero
*L*_6_–*C*_6_	11	0.209341	Auxiliary upper-stopband zero
*L*_7_–*C*_7_	17	0.087648	Auxiliary upper-stopband zero

**Table 3:** Fixed LTCC and HFSS parameters used in the physical model.

Parameter	Value	Role in the Model
Relative permittivity, *ε*_r_	7.0	LTCC dielectric
Dielectric loss tangent	0.002	LTCC dielectric loss
Individual dielectric layer thickness	60 µm	Multilayer stack
Via diameter	100 µm	Fabrication constraint
Minimum metal-line width	100 µm	Fabrication constraint
Conductor thickness	10 µm	Metal model
Conductor material	Silver	Metal model
Active filtering footprint	2.8 mm × 2.3 mm	Filter network
Overall packaged size	4 mm × 2.8 mm × 1 mm	Packaged device

**Table 4:** Normalized first-order sensitivity used to assess the dominant electrical variations.

Perturbed Quantity	Perturbation	Response Quantity	Calculated Change	Interpretation
L or C	+5%/−5%	Resonance frequency	−2.41%/+2.60%	Normalized resonator sensitivity
*ε*_r_ (with C ∝ *ε*_r_)	+5%/−5%	Resonance frequency	−2.41%/+2.60%	Dielectric-permittivity sensitivity
Dielectric thickness t (C ∝ 1/t)	+5%/−5%	Resonance frequency	+2.47%/−2.53%	MIM-capacitance sensitivity
Mutual inductance M	+5%/−5%	Coupling coefficient k	+5.00%/−5.00%	Proxy for overlap/misalignment variation
*L*_1_ and *L*_2_ together, M fixed	+5%/−5%	Coupling coefficient k	−4.76%/+5.26%	Self-inductance sensitivity
Conductor width	Geometry-dependent	Effective L and conductor loss	Represented by normalized L sensitivity	First-order electrical representation

**Table 5:** Comparison with representative reported bandpass filters.

Ref.	Technology/ Order	*f*_0_ (GHz)	3 dB FBW (%)	IL (dB)	TZs	Stopband Rejection/ Normalized Extent	Normalized Footprint (×10^−3^ *λ*_0_^2^)	Principal Implementation Feature
[2]	LTCC/3rd	3.5	32	1.35	5	−20 dB @ 2.2 *f*_0_	5.47	Stepped-impedance stubs
[3]	LTCC/4th	N/R	N/R	1.3	3	>40 dB; extent N/R	N/R	Cross-coupled LTCC resonators
[13]	LTCC/3rd	3.9	45	0.76	4	−27 dB @ 5.7 *f*_0_	0.224	Tri-mode lumped resonators in heterogeneous LTCC
[14]	GaAs IPD/3rd	3.9	43.3	1.63	2	−20 dB @ 6.34 *f*_0_	0.41	IPD coupling scheme
This work	LTCC/5th	4.0	50	1.35 (sim.)	4	>30 dB, DC–2 and 6–18 GHz; upper limit 4.5 *f*_0_	1.17 *	FDC ladder, mutually coupled inductors, and auxiliary FDC poles

* For the proposed filter, the normalized footprint is calculated from the 2.8 mm × 2.3 mm active filtering area at *f*_0_ = 4 GHz; the overall packaged size is 4 mm × 2.8 mm × 1 mm. N/R: Not reported in the cited reference.

## Data Availability

The original contributions presented in this study are included in the article. Further inquiries can be directed to the corresponding authors.
